# Colney Hatch Asylum
*A Letter to the Committee of Visitors of the Additional Lunatic Asylum, Colney Hatch, in reference to the Contemplated Constitution of the Medical Staff, Thomas Stone, M.D., Fell, of the Med. Chi. Soc. &c.


**Published:** 1850-07-01

**Authors:** 


### COLNEY HATCH ASYLUM.*

[We had proposed entering at some length into the important question connected with the organization of the medical staff of our great national establishments for the care and treatment of the insane. This is a subject which loudly calls for discussion; at present, however, we decline, for reasons which will be obvious to our readers, saying anything on the subject. The following observations are published for private circulation by *Dr. Thomas Stone*, in the form of a letter addressed to the Committee of Visitors. We append a copy of this able production for the perusal of those who may not have seen it in its original form.*]

* A Letter to the Committee of Visitors of the Additional Lunatic Asylum, Colney Hatch, in reference to the Contemplated Constitution of the Medical Staff, Thomas Stone, M.D., Fell. of the Med. Chi. Soc. &c.

A public announcement having appeared in the daily newspapers in reference to the constitution of the Medical Staff of the New Lunatic Asylum for Middlesex, Colney Hatch, we trust we may, without incurring the imputation of officiously interfering with the functions of the County Magistracy, venture to offer a few observations in reference to the proposed arrangements.

A very heavy responsibility is now resting upon the Magistracy—in whose hands are entrusted the general and medical organization of one of the largest County Lunatic Asylums ever erected in this country. It remains with them to decide whether the many thousand helpless and insane poor who, in all probability, will, in a few years, be crowded into this Asylum, shall be efficiently treated or not; whether they shall enjoy the advantages which may be derived from the present advanced state of this branch of medical science, or be left to pine away their miserable existence in a state of hopeless and incurable insanity.

The architectural arrangements for Colney Hatch, it would appear, have been made for the reception of not less than a thousand patients; and the general impression on the public mind, and that of the profession, is, that the Magistrates, actuated by a highly liberal and philanthropic spirit, are anxious to render the present Asylum not merely a receptacle or *house of detention* for the insane, but a *curative establishment*, which shall, while it ministers to the relief of suffering humanity, do something to advance the interests of science. It is a fact—which is well known to the profession—that the progress of pathology has thrown light upon the nature and treatment of the most obscure diseases of the chest and abdomen, because the public hospitals have provided for general diseases a clinical school in connexion with them; but our County Lunatic Asylums—which are, in point of fact, hospitals for mental disease—are left with so inefficient a medical staff, as to render any analogous pathological researches in connexion with cerebral disease almost impracticable. When we look to the constitution of the Salpêtrière, the Bicêtre, Charenton, and other Lunatic Asylums on the Continent, we find a full complement not only of resident medical officers, but extra domiciled consulting Physicians; and surely the Metropolitan County ought not to lag so miserably behind the spirit of progression as to provide only for the safe custody of insane poor, overlooking entirely the means so palpably within reach of advancing medical knowledge, and thus conducing to improvement in the treatment and the permanent cure of the disease.

With these views, the question for the consideration of the Magistrates is, whether the medical arrangements as developed in the subjoined advertisement* are such as will carry out the original

* MIDDLESEX ADDITIONAL COUNTY ASYLUM, Colney Hatch.—WANTED, TWO RESIDENT MEDICAL OFFICERS for the above Asylum, which it is expected will be ready for the reception of Patients by the end of the present year or the beginning of 1851. They must be Doctors of Medicine, Fellows or Licentiates of the Royal College of Physicians of London, Edinburgh, intention of the Committee, and at the same time give satisfaction to the profession and the public? We are apprehensive that neither of these objects will be attained. In the first place, as to the qualifications of the candidates. It is not to be expected that many members of the profession possessing the amount of qualification specified, who have enjoyed that enlarged degree of practical experience in the management and treatment of the insane which can alone fit them for the efficient discharge of the responsible duties that will devolve upon them, will come forward as candidates for such a post. It is the duty of the Magistracy to look principally to the professional knowledge and moral character of the candidates. The mere possession of diplomas indicative of the holder being a member of this or that medical corporation, of his possessing this or that degree in surgery or medicine, ought to be esteemed as only minor elements in the great question at issue. We do not maintain that the degrees or honours of the medical candidates are not to be well weighed in the scale, but they ought to be held secondary to other and more vitally important considerations. High moral character, firmness, and energy of mind, suavity of manner, solidity of judgment, a heart ever alive to the sufferings of our fellow-men, combined with a practical knowledge of the treatment of insanity and the requirements of the insane, are qualifications so indispensable, that the possession of all degrees, and fellowships, and academical honours, in medicine, surgery, and pharmacy, which it may be in the power of our first universities to confer, ought not for one moment to be placed in competition with them.

We do not wish our view upon this matter to be misunderstood. Qualifications are necessary. The magistrates have a right to demand that those offering themselves for so important a trust should produce evidence of having enjoyed the advantages of a regular education; but practical experience in the treatment and management of the insane can alone indicate the general fitness of any candidate to discharge the duties of such an office. The public advertisement, we regret to say, conveys a different idea of the intentions of the magistracy. In the present state of the profession, medical and surgical degrees are not the safest and best guarantees of professional or Dublin, or Members or Fellows of the Royal College of Surgeons, and in either case Members or Licentiates of the Apothecaries' Company. The salary of each will be 200*l*. per annum, with board and allowance of coals and candles. Candidates are requested to send in their Testimonials, addressed to the "Committee of Visitors of the Colney Hatch Lunatic Asylum," before the 2nd of July next, under cover to me at the Sessions House, Clerkenwell, and to attend the Committee at that place on Wednesday, the 3rd day of July next, at Two o'clock precisely.

JOHN S. SKAIFE, Clerk to the Committee,
Sessions House, Clerkenwell, May 7th, 1850.

This advertisement renders it imperative that candidates shall be Licentiates of the Apothecaries' Company, yet Physicians, whether graduates of Oxford, Cambridge, Edinburgh, Dublin, are by the very course of their education precluded from belonging to the Apothecaries' Company.

status or qualification. For the last six or seven years we have been on the eve of great changes in the medical and surgical corporate bodies; and as nearly all the public colleges have applied to the Government for new charters of incorporation, medical men, anxious to possess the highest degree—medical and surgical—have held back, not for want of the necessary knowledge, but until the question of medical reform shall be settled by the government.

But to the point at issue. Supposing the Committee of Visitors are able to find two fully qualified men to fill the vacant posts, will they be sufficient to discharge the duties devolving upon them? Remember, there will be 500 patients, at least, intrusted to the care of each medical officer. Is it possible that these poor insane persons can receive a tithe of the attendance due to them? If cure be no object—if the medical superintendent be required simply to walk through the wards mechanically every day, to enter the names of the patients as they present themselves at the establishment, then it is possible all may go on smoothly and satisfactorily; but surely there are other, and more sacred duties, which will devolve upon the Resident Medical Officers of the Colney-Hatch Institution. If patients are to be treated according to the most improved method, it will be impossible for the medical officer to examine with that degree of minuteness each individual case, for the purpose of applying resources of remedial art to the number of patients he will have constantly under his care. Those unfortunate persons, therefore, must of necessity be cursorily looked to, however anxious the medical officer may be to adopt a curative process of treatment. Independently of his purely professional, let us look at his collateral duties—what will they be? He will be required, by the provisions of the Act of Parliament, to attend to all certificates of admissions and discharges, sending in copies of each to the Commissioners in Lunacy; he will be required to enter these in the registers prescribed, and also keep up his weekly reports; he will, furthermore, be required to enter into a case-book a minute history of each case, detailing its origin, early symptoms, progress, treatment, &c.; and if the medical officers are to discharge their duties efficiently, it will be imperative upon them to institute, with care, a post-mortem examination of all who die in the Asylum. These autopsies, if conducted in the spirit of modern science, will occupy a considerable portion of the Resident Medical Officer's time; for, in order to appreciate correctly the pathological changes effected in the brain and nervous system, he must make a patient microscopical examination of the delicate nervous structure, in order to detect those minute pathological appearances which are unappreciable to the naked eye, in which way only can any satisfactory results be obtained. In addition to all this, the candidate will be required to attend the meetings of Magistrates on Board days; and furthermore, if the establishment is to be rendered useful in a scientific and educational point of view, he will be called upon to preside over a school of Mental Pathology, and communicate to his class the results of his observations. How, in common sense, can the two medical men, having, day and night, charge of a thousand patients, accomplish such multifarious duties conscientiously to themselves, satisfactorily to the patients, and beneficially to the poor unfortunate inmates?

Again: In addition to these two Resident Physicians, is it not contemplated to appoint any Consulting or Visiting Physicians to so large an Asylum? Is the Medical Staff to be restricted to only *two* medical officers? Surely the magistrates cannot suppose the county at large will be satisfied with this arrangement. There is not a Hospital or Dispensary in the metropolis, or in any town in the United Kingdom, that has not its Consulting Physicians in addition to the ordinary Medical Staff; and we can well conceive the practical advantages consequent upon this principle, which has been acted upon in the organization of all such public Institutions, both at home and abroad. Independently of the great relief which such an appointment would afford to the Resident Medical Officers, from the fact of their having at command, in all cases of difficulty and emergency, a physician of eminence and experience to confer with, there are other reasons which entitle this question to serious consideration. There always will be, in the capital of every country, and in the leading provincial towns, men who have devoted, at a great personal sacrifice, their time and ability to the study of special diseases. These men are entitled to some public recognition of their *status*, and the only reward which they can look forward to, is being appointed, as an honorary distinction, to the public Medical Charities and other Institutions of the country. If this legitimate object of their ambition be taken from them, a manifest injustice will be inflicted upon many of the most accomplished and meritorious members of the profession. In conclusion, we would observe, that in so influential and affluent a county as that of Middlesex, it is to be hoped that the Magistrates will not be influenced by mistaken notions of economy, but organize such an Asylum as Colney-Hatch promises, and ought to be, upon such a liberal scale as will render it a model establishment, deserving of imitation in this and other countries.

